# Supplementary material for: Antimicrobial activity of natural quinone-methide triterpenoids celastrol and pristimerin against pathogenic Neisseria
Source: Microbiol Spectr. 2025 Dec 30;14(2):e02724-25. doi: 10.1128/spectrum.02724-25 (PMC12889087; doi:10.1128/spectrum.02724-25)
Supplement: Supplemental material — Dataset S1: List of 54 chemically defined natural compounds from NatureBank library. Method S1: UHPLC-MS compound preparation and purity analysis conditions. Figure S1: UHPLC-MS analysis of celastrol. Figure S2: UHPLC-MS analysis of pristimerin. Table S1: MIC and MBC of CL, PR, AZ, and CRO in Ng. [file spectrum.02724-25-s0001.docx]

**Antimicrobial Activity of Natural Quinone-Methide Triterpenoids Celastrol and Pristimerin Against Pathogenic *Neisseria***

Alice Ascari ^a^, Taha ^a^, Rohan A. Davis ^a-c^, Kate L. Seib ^a,1^ and Evgeny A. Semchenko ^a,1,*^

^a^ Institute for Biomedicine and Glycomics, Griffith University, Parklands Drive, Gold Coast, Queensland, 4222, Australia

^b^ School of Environment and Science, Griffith University, Kessels Road, Nathan, Queensland 4111, Australia

^c^ NatureBank, Griffith University, Kessels Road, Nathan, Queensland 4111, Australia

**CORRESPONDANCE**

^*^ Evgeny A. Semchenko; [e.semchenko@griffith.edu.au](mailto:e.semchenko@griffith.edu.au)

**Supplementary data contents**

**Dataset S1**. List of 54 chemically defined natural compounds from NatureBank library.

**Method S1.** UHPLC-MS compound preparation and purity analysis conditions.

**Figure S1.** UHPLC-MS analysis of celastrol.

**Figure S2.** UHPLC-MS analysis of pristimerin.

**Table S1.** Minimum Inhibitory Concentration (MIC) and Minimum Bactericidal Concentration (MBC) of celastrol, pristimerin, azithromycin and ceftriaxone in *N. gonorrhoeae* 1291, WHO X and WHO X Δ*mtrE*.

**Dataset S1**. List of 54 chemically defined natural compounds from NatureBank library (1, 2).

Microthecaline A; Pestalactam A; Anopterine; (-)-Xylariamide A; Psammaplysin F; Thiaplakortone A; Thiaplakortone B; Bilocularin A; Bilocularin B; Anibadimer A; Serrulatane analogue A; Serrulatane analogue B; Serrulatane analogue C; 3-chloro-4-hydroxyphenylacetamide, 3-chloro-4-hydroxyphenylacetic acid, Endiandrin A; Endiandrin B; (-)-dihydroguaiaretic acid; Entonalactam A; Entonalactam B; Pestalactam D; Celastrofuran E; Denhaminol A; Pristimerin; Celastrol; Debromohymenialdisine; Hymenialdisine; Dibromophakelline; Melicopidine; Tambjamine F; Tambjamine C; Gibberellic acid; Artemisinin; Boldine; Papaverine N-oxide; Papaverine; 2'-Chloropapaverine; 2'-Chloropapaverine N-oxide; Goniothalamin; D/L-Kavain; Desmethoxyyangonin; Dihydrokavain; Yangonin; Dihydromethysticin; Methysticin; Acanthophorin A; Acanthophorin B; Epiisoshinanolone; Aloenin A; Cynaropicrin; Fuziline; Huperzine A; Lithospermic acid; Paeoniflorin

**Method S1.** UHPLC-MS compound preparation and purity analysis conditions. Celastrol and pristimerin and were both weighed out (1.0–2.0 mg) and dissolved in MeOH (1.0–2.0 mL) to generate a stock solution, which had a final concentration of 1 mg/mL. The UHPLC-MS analysis was performed on an Ultimate 3000 RS UHPLC coupled to a Thermo Fisher Scientific MSQ Plus single quadruple ESI mass spectrometer using a Thermo Scientific Accurose C_18_-bonded silica column (2.6 μm, 80 Å, 150 × 2.1 mm). All compounds (1 mg/mL, 100% MeOH) were subjected to UHPLC-MS profiling using the Accurose analytical column with an injection volume of 5 µL. Isocratic conditions of 10% MeOH/90% H_2_O (0.1% formic acid) were employed for the first minute, followed by a linear gradient to 100% MeOH (0.1% formic acid) over 8 min, then an isocratic elution of 100% MeOH (0.1% formic acid) for 1.5 min, before returning to 10% MeOH/90% H_2_O (0.1% formic acid) in 0.25 min followed by re-equilibration of the column using  isocratic conditions of 10% MeOH/90% H_2_O (0.1% formic acid) for 2.25 min all at flow rate of 0.3 mL/min. UHPLC-MS data was analysed using Thermo Scientific^TM^ Dionex^TM^ Chromeleon^TM^ 7 Chromatography data system (Version 7.2.10) at a wavelength of 254 nm.

**Figure S1.** UHPLC-MS analysis of celastrol. (a) UV chromatogram recorded at 254 nm shows a single dominant peak at 12.24 min. No other significant peaks were observed, suggesting a purity >95% based on visual inspection under the applied chromatographic conditions. (b) Mass spectrum (+ve mode) at the peak apex (12.24 min) displays major ions at *m/z* 451.3 and 452.4, consistent with the expected pseudomolecular ion of [M+H]^+^ for celastrol.


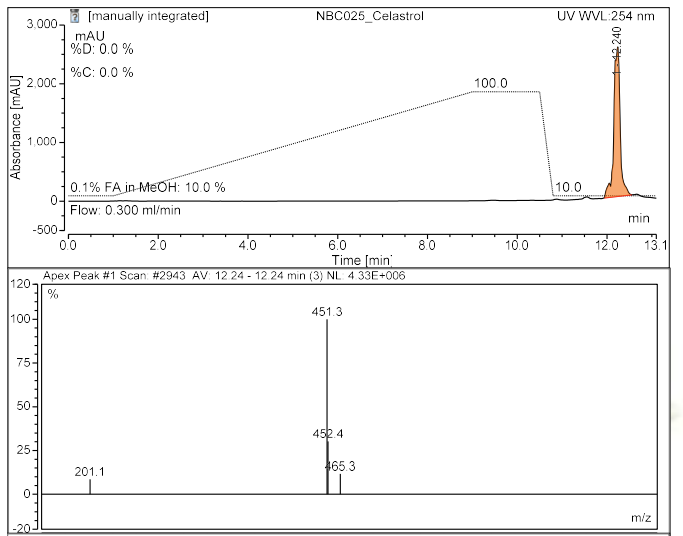


**(a)**

**(b)**

**Figure S2.** UHPLC-MS analysis of pristimerin. (a) UV chromatogram recorded at 254 nm shows a single dominant peak at 12.6 min. No other significant peaks were observed, suggesting a purity >95% based on visual inspection under the applied chromatographic conditions. (b) Mass spectrum (+ve mode) at the peak apex (12.60–12.61 min) displays major ions at *m/z* 465.3 and 466.4, consistent with the expected pseudomolecular ion of [M+H]^+^ for pristimerin.


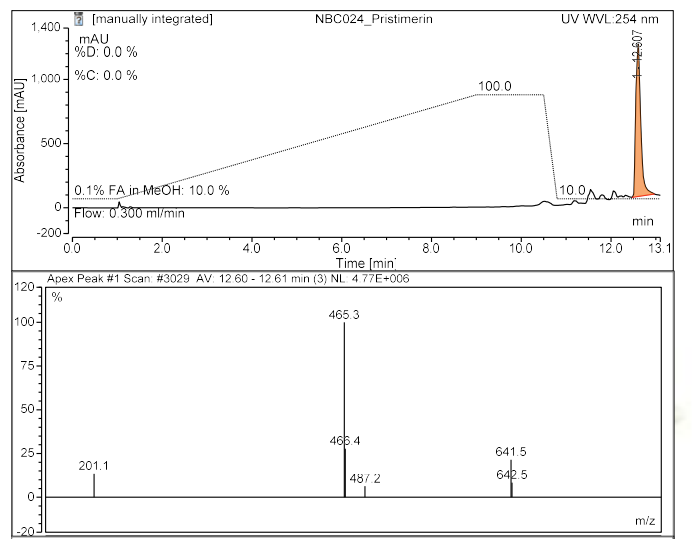


**(a)**

**(b)**

**Table S1.** Minimum Inhibitory Concentration (MIC) and Minimum Bactericidal Concentration (MBC) of celastrol, pristimerin, azithromycin and ceftriaxone in *N. gonorrhoeae* 1291, WHO X and WHO X Δ*mtrE*.

| **Strain** | **Azithromycin** | **Ceftriaxone** | **Celastrol** | | **Pristimerin** | | |
| --- | --- | --- | --- | --- | --- | --- | --- |
|  | **MIC**^*^ **/ sensitivity**^†^ | **MIC / sensitivity** | **MIC** | **MBC** | | **MIC** | **MBC** |
| 1291 | 0.031 (S) | 0.004 (S) | 2.8 | 0.7 | | 2.9 | 5.8 |
| WHO X | 0.5 (S) | 2 (HLR) | 1.4 | 5.6 | | 5.8 | 5.8 |
| WHO X Δ*mtrE* | 0.031 (S) | 1 (R) | 0.7 | 2.8 | | 1.5 | 1.5 |

^*^ MIC was determined using broth microdilution as described in Materials and Methods (3).

^†^ Antimicrobial sensitivity: S – susceptible; R – resistant; HLR - high level resistance. Azithromycin sensitivity as determined by epidemiological cut-off of MIC >1mg/L as no clinical breakpoints are reported by EUCAST. Ceftriaxone sensitivity as per EUCAST guidelines ([www.eucast.org/clinical_breakpoints](http://www.eucast.org/clinical_breakpoints)).

**References**

1. NatureBank. Griffith University. <https://www.griffith.edu.au/research/institute-biomedicine-glycomics/facilities/naturebank>. Accessed September 2025.

2. Macdonald JR, Fisher GM, Hayes S, Skinner-Adams TS, Davis RA, Andrews KT. 2025. Antiplasmodial activity of tambjamines, dihydro-beta-agarofurans and pyrroloazepines derived from Australian plant and marine species. Bioorg Med Chem Lett 129:130369.

3. Evert BJ, Slesarenko VA, Punnasseril JMJ, Taha, Zhan J, Zhou Y, Semchenko EA, Seib KL. 2022. Self-Inhibitory Peptides Targeting the *Neisseria gonorrhoeae* MtrCDE Efflux Pump Increase Antibiotic Susceptibility. Antimicrob Agents Chemother 66:e0154221.
